# “[It] is now my responsibility to fulfill that wish:” Clinical and rapid autopsy staff members’ experiences and perceptions of HIV reservoir research at the end of life

**DOI:** 10.1371/journal.pone.0242420

**Published:** 2020-11-18

**Authors:** Kelly E. Perry, Jeff Taylor, Hursch Patel, Sogol Stephanie Javadi, Kushagra Mathur, Andy Kaytes, Susanna Concha-Garcia, Susan Little, Davey Smith, Sara Gianella, Karine Dubé

**Affiliations:** 1 UNC Gillings School of Global Public Health, Chapel Hill, NC, United States of America; 2 AntiViral Research Center Community Advisory Board, San Diego, CA, United States of America; 3 HIV + Aging Research Project–Palm Springs (HARP-PS), Palm Springs, CA, United States of America; 4 AntiViral Research Center (AVRC), University of California San Diego, San Diego, CA, United States of America; 5 HIV Neurobehavioral Research Center, University of California San Diego, San Diego, CA, United States of America; 6 Division of Infectious Diseases and Global Public Health, University of California San Diego, San Diego, CA, United States of America; Universitat d'Alacante, SPAIN

## Abstract

**Introduction:**

Little is known about the effects of HIV reservoir research at the end of life on staff members involved. Staff members’ perceptions and experiences were assessed related to their involvement in the Last Gift, a rapid autopsy study at the University of California San Diego enrolling people living with HIV who are terminally ill and have a desire to contribute to HIV cure-related research.

**Methods:**

Two focus group discussions consisting of clinical (*n* = 7) and rapid research autopsy (*n* = 8) staff members were conducted to understand the perspectives of staff members and the impact the Last Gift rapid autopsy study had on them. The total sample consisted of 66.7% females and 33.3% males and was ethnically diverse (66.7% Caucasian, 6.7% African American, 20.0% Asian descent, 6.7% Hispanic descent and American Indian) with a range of experience in the HIV field from 1 year to 30 years.

**Results:**

Qualitative focus group data revealed five major themes underlying study staff members’ multilayered mental and practical involvement: 1) positive perceptions of the Last Gift study, with sub-themes including Last Gift study participants’ altruism, fulfillment, and control at the end of life, 2) perceptions of staff members’ close involvement in the Last Gift study, with sub-themes related to staff members’ cognitive processing, self-actualization and fulfillment, stress management and resilience, coping mechanisms, and gratitude toward Last Gift participants and toward the study itself, 3) considerations for successful and sustainable study implementation, such as ethical awareness and sustained community and patient engagement, 4) collaborative learning and organizational processes and the value of interdependence between staff members, and 5) considerations for potential study scale-up at other clinical research sites.

**Discussion:**

Understanding staff members’ nuanced emotional and procedural experiences is crucial to the Last Gift study’s sustainability and will inform similar cure research studies conducted with people living with HIV at the end of life. The study’s potential reproducibility depends on a robust research infrastructure with established, interdependent clinical and rapid autopsy teams, continuous community engagement, and an ethical and well-informed engagement process with people living with HIV.

## Introduction

Guidelines on HIV reservoir research at the end of life (EOL) are emerging, though little is known about effects of such research on staff members involved [1, 2]. The Last Gift is a rapid autopsy research study at the University of California San Diego (UCSD) enrolling people living with HIV (PLWHIV) who are terminally ill and have a strong desire to contribute to HIV cure-related research [3–5]. Participants must have an advanced or terminal illness with a prognosis of less than 6 months in order to be eligible for the study. The Last Gift study does not involve palliative care and confers no expectation of direct clinical benefits nor prospect of being cured of HIV or the terminal illness [1].

Last Gift participants undergo blood draws and optional biological sample collections to characterize HIV reservoirs–locations where HIV remains latent in the body–as well as socio-behavioral assessments about their experiences participating in an HIV cure-related research study at the EOL. The participants and their next-of-kin/loved ones frequently interact with the study’s clinical team during these biological and socio-behavioral procedures. The primary post-mortem procedure is a full body donation as well as a rapid research autopsy performed by a separate team within 6 hours of death [2]. The precedent that inspired and underpinned our research exists in the field of oncology [6].

Due to the novel and potentially controversial nature of EOL HIV cure-related research, we are conducting extensive empirical research with Last Gift participants and their next-of-kin/loved ones to understand their perceptions of such research [7, 8]. To complement these perspectives, we implemented focus groups in order to understand the perspectives of clinical and rapid research autopsy staff members and the impact that the Last Gift study had on them. Implementation of these focus groups was motivated by three main factors: 1) EOL HIV cure research is novel and perceptions of all those involved (including staff members) in such research is valued for continued effective study implementation and potential future scale-up and sustainability, 2) the effects that EOL HIV cure research may have on staff members (such as emotional impact) involved have yet to be understood, especially given the dearth of articles published on perceptions of research staff, and 3) focus group discussions provided staff members the opportunity to debrief about the study as teams. In this paper, we report the perceptions and experiences of the Last Gift clinical and rapid autopsy team members and their involvement in EOL HIV cure-related research. A qualitative approach was utilized to elicit perspectives around a sensitive topic (e.g. EOL research, rapid autopsy processes, etc.) and to conduct formative research on a topic about which very little is known, particularly the perspectives and experiences of staff involved in EOL HIV cure-related research.

## Methods

The Last Gift study was approved by the UCSD Institutional Review Board (IRB#190519SX ‘Last Gift Study Focus Groups’). Clinical and rapid research autopsy staff provided written informed consent to be part of a focus group as a separate component of the main Last Gift clinical study protocol. Focus group discussion routes (Table 1) were developed in close collaboration with the AntiViral Research Center (AVRC) Community Advisory Board and the Palm Springs Positive Life Program. Research staff did not receive compensation for their time.

**Table 1:** Focus group discussion routes for clinical and rapid autopsy staff involved in the Last Gift study.

**Introductory/General Questions**
• First, thank you so much for your time.
• What does the Last Gift study mean to you?
• What do you think the Last Gift study means/meant to the study participants?
**Focus Group Discussion Questions for Clinical Staff Involved in the Last Gift Study**
• What does it mean to you to be part of the study as a staff member?
• What are your feelings about the Last Gift study?
• Has the Last Gift study changed your life? If yes, how so?
• Do you develop a bond with the Last Gift study participants? Can you please explain?
• How do you feel when a Last Gift study participant passes away?
• Is there anything you wish you had known before your involvement with the Last Gift study? If so, what is it?
• Do/did you see any benefits/positives to the Last Gift study participants of being in the study?
• Do/did you see any risks/negatives to the Last Gift study participants of being in the study?
Resilience/Coping
• How do you/did you manage the stress associated with the Last Gift study?
• Have you experienced any emotional issue (i.e. burden, burn out) as a result of the study?
• In what ways do you cope with the feelings involved in the study?
Ethical Considerations
• Do you see any ethical issue with the Last Gift study?
• We often worry that people who are terminally ill are a vulnerable population. Do you consider the Last Gift study participants to be a vulnerable group?
• Right now, the Last Gift study has been observational, looking at HIV reservoirs inside the body of the study participants. How would you feel about introducing HIV cure-related research interventions?
◦ Can you name any ethical considerations for testing broadly neutralizing antibodies in this population?
◦ Can you name any ethical considerations for testing chimeric antigen receptor T cells (CAR T) cells in this population?
**Focus Group Discussion Questions for Staff Involved in the Last Gift Rapid Autopsy Process**
• What does it mean to you to be part of the study as a staff member?
• What are your feelings about the Last Gift study?
• Has the Last Gift study changed your life? If yes, how so?
• Is there anything you wish you had known before your involvement with the Last Gift study? If so, what is it?
Autopsy Process
• Can you please describe the rapid autopsy process?
• What does the rapid autopsy process mean to you?
• Do you have any fear associated with seeing dead people during the rapid autopsy process?
Resilience/Coping
• How do you/did you manage the stress associated with the Last Gift study?
• Have you experienced any emotional distress as a result of the study? If yes, can you please describe?
• In what ways do you cope with the feelings involved with the Last Gift study?
Ethical Considerations
• Do you see any ethical issue with the Last Gift study?
• Right now, the Last Gift study has been observational, looking at HIV reservoirs inside the body of the study participants. How would you feel about introducing HIV cure-related research interventions?
◦ Can you name any ethical considerations for testing broadly neutralizing antibodies in this population?
◦ Can you name any ethical considerations for testing CAR T cells in this population?
**Ending Questions**
• Do you have any recommendation to improve the conduct of the study?
• Can you think of anything else you would like to share with the group on this topic?

In May 2019, trained facilitators (S.L. and K.D.) conducted two focus group discussions: 1) one with the Last Gift study’s clinical team, and 2) one with the study’s rapid research autopsy team. Focus groups were conducted in-person in a conference room, using prescribed probes as necessary. A research assistant (S.S.J.) took detailed notes. Focus group discussions covered the following topics: staff members’ perceptions and experiences pertaining to the Last Gift study, resilience and coping mechanisms, as well as ethical considerations. All questions were open-ended, and each focus group discussion lasted approximately 90 minutes.

After focus group discussions were completed, audio files were uploaded into the secure study database (RedCap, Vanderbilt University, TN), which were then transcribed to Microsoft Word with personal identifiers removed by research staff (K.P.). Transcripts were reviewed by a second research staff (H.P.) for quality control. After transcription and quality control were completed, the original audio files were deleted from the secure server, as indicated in the IRB application.

Focus group discussion data were double-coded manually (by K.P. and K.D.) into emergent themes using an inductive approach [9]. Due to the novelty of the topic, we did not use a pre-existing coding scheme. We applied conventional content analysis to organize text units into a structured format. Our analytical methods were inspired by the phenomenological approach, which aided in characterizing a novel phenomenon, particularly as no prior literature on the effects of rapid research autopsy on staff existed [9]. Key emergent themes and associated quotes were organized into a Microsoft Word processing document. The most prominent quotes reflecting our key themes can be found verbatim in the Results section. Quotes were not ascribed to staff participants due to the nature of conducting focus groups, but respective focus groups from which quotes originated from are noted. Supplementary quotes are included as a supplement to this manuscript (S1 Appendix).

## Results

### Demographic characteristics of Last Gift clinical and rapid autopsy team members

Focus groups consisted of the Last Gift clinical (*n* = 7) and rapid research autopsy (*n* = 8) staff members. The total sample consisted of 15 clinical and autopsy team members (66.7% females; 33.3% males) and was ethnically diverse (66.7% Caucasian, 6.7% African American, 20.0% Asian descent, 6.7% Hispanic descent and American Indian) (**Table 2**) with a range of experience in the HIV field from 1 year to 30 years (mean: 7 years). Participants’ highest degrees obtained ranged from Bachelors of Science and Arts, Master of Science, Doctor of Medicine, and Doctor of Philosophy.

**Table 2:** Demographic variables of Last Gift clinical and rapid autopsy team members (San Diego, California, May 2019).

Focus Group Type	Gender/Sex	Race/Ethnicity	Role in Last Gift Study
Clinical Team	Female	Hispanic Descent & American Indian	Staff member
Clinical Team	Female	Caucasian/Non-Hispanic	Staff member
Clinical Team	Male	Caucasian/Non-Hispanic	Staff member
Clinical Team	Male	Caucasian/Non-Hispanic	Staff member and Rapid autopsy
Clinical Team	Female	Caucasian/Non-Hispanic	Staff member
Clinical Team	Male	Caucasian/Non-Hispanic	Community representative
Clinical Team	Male	Caucasian/Non-Hispanic	Staff member and Rapid autopsy
Autopsy Team	Female	Caucasian/Non-Hispanic	Staff member and Rapid autopsy
Autopsy Team	Female	Caucasian/Non-Hispanic	Staff member and Rapid autopsy
Autopsy Team	Female	Black/African-American	Staff member and Rapid autopsy
Autopsy Team	Female	Asian Descent	Staff member and Rapid autopsy
Autopsy Team	Female	Asian Descent	Rapid autopsy
Autopsy Team	Female	Caucasian/Non-Hispanic	Staff member and Rapid autopsy
Autopsy Team	Male	Caucasian/Non-Hispanic	Staff member and Rapid autopsy
Autopsy Team	Female	Asian Descent	Rapid autopsy

**Fig 1:**
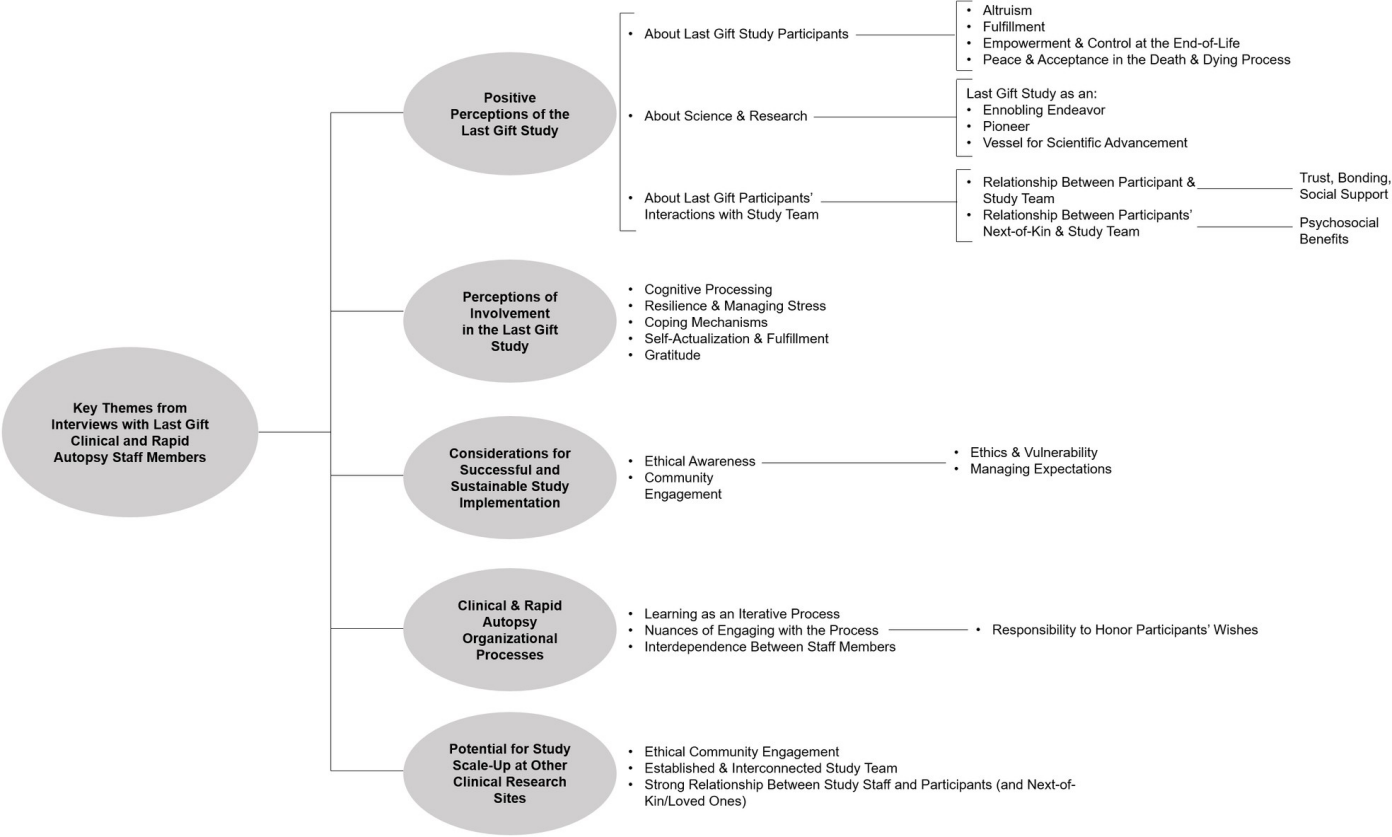
Analytic coding tree from focus group discussions with Last Gift clinical and rapid autopsy team members and implications for EOL HIV cure research (San Diego, California, May 2019).

Our qualitative focus group data revealed five major themes: 1) positive perceptions of the Last Gift study, with sub-themes related to Last Gift study participants’ altruism, fulfillment, and control at the EOL, among others, 2) perceptions of staff members’ close involvement in the Last Gift study, with sub-themes harkening on staff members’ cognitive processing, stress management and resilience, coping mechanisms, self-actualization and fulfillment, and gratitude towards participants and the study, 3) considerations for successful and sustainable study implementation, with sub-themes centered on the need for ethical awareness and sustained community and patient engagement, 4) clinical and rapid autopsy staff’s organizational processes, with sub-themes highlighting learning processes and interdependence between staff members, and 5) considerations for potential study scale-up at other clinical research sites, with sub-themes related to established study teams and strong relationships between staff and participants. These key themes were common between the clinical and rapid autopsy team members and converged upon similar focal points and sub-themes without major divergence. For instance, the clinical team more saliently highlighted the Last Gift study participants’ altruism, fulfillment, and control at the EOL because clinical team staff were more directly involved with the study participants during their terminal illness compared to rapid autopsy staff members. Rapid autopsy staff also recognized study participants’ altruism, though they nested this sentiment within their overarching articulation of gratitude toward study participants, as described below. An analytic coding tree of key themes from focus group interviews is outlined in **Fig 1**.

### Positive perceptions of the Last Gift study

#### Perceptions about Last Gift study participants

Clinical team members shared similar perceptions of the Last Gift study, articulating four primary themes centered around Last Gift study participants: 1) deep sense of altruism, 2) fulfillment to be part of research, 3) empowerment and control at the EOL, and 4) peace and acceptance with the death and dying process–all as expressed or demonstrated by the study’s participants. Because rapid autopsy staff members are not as involved with Last Gift study participants during the dying process, they did not share many quotes that were relevant to this theme.

Regarding altruism, a clinical team member expressed:

*This [study] is something [participants] are doing for unselfish reasons to help others around them who aren’t here because of living with HIV and*, *um*, *[…] that they want to make sure that they also are a part of the solution or as one of our participants said*, *‘part of the puzzle’*. (Clinical Team Focus Group)

A rapid autopsy team member echoed these sentiments:

*[T]o our participants*, *[the study] does mean a lot*, *because really it’s their last act is to donate themselves for the benefit of others for hope of maybe finding a cure*, *for hope of maybe helping others live a better life*, *and…so I think it’s something that*, *you know*, *that they really believe in*, *in order for them to be able to do this*. (Rapid Autopsy Team Focus Group)

Clinical team members expressed that Last Gift participants experienced fulfillment as a result of study involvement. For example, they noted:

*One participant […] thought that no one would want his body*, *and that he wasn’t valuable to anybody…knowing that this study was going to find value in his body…his death really brought that participant alive*. (Clinical Team Focus Group)

*Each individual that I speak to has told me that it makes their death and their life well worth living*. *That they feel they’re doing this for others*. (Clinical Team Focus Group)

Additionally, the clinical team described empowerment and control at the EOL as displayed saliently by Last Gift participants. For example, members of the clinical team expressed:

*I have not seen a study where participants have felt so empowered*. (Clinical Team Focus Group)

*Most of these patients have no control over their dying process*, *and this gives them some control*, *some choice over some of their final decisions*. (Clinical Team Focus Group)

Clinical team members recounted their interactions with Last Gift participants and next-of-kin/loved ones, specifically highlighting participants and loved ones’ perceptions of the study as nested within the death and dying process.

*[N]ext-of-kin really said it loud and clear that the study participants actually had an easier time dying knowing that they were part of the Last Gift [study]*, *rather than just dying and that was it*. *I think it means really a lot for people to have this opportunity*. (Clinical Team Focus Group)

*[D]espite being a physician for a whole lot of years and seeing a whole lot of people die*, *I’m not used to seeing so many people die content…satisfied*, *comforted*, *with what their final days are*. (Clinical Team Focus Group)

Clinical team members also described Last Gift participants’ death and dying process through personal biomedical and psychosocial lenses:

*[I]t’s where the personal and professional meet in medicine*. *I think every doctor probably remembers most of the deaths that he or she has presided over*, *or*, *you know*, *signed off on*. *[…] I remember patients that*, *I*, *you know*, *um*, *signed their death certificates as a resident*, *but it’s on that same list that the Last Gift patients are for me*, *but even stronger because I continue to deal with their charts*. (Clinical Team Focus Group)

*I personally feel relief*, *and I know that’s kind of morbid*, *but*, *like*, *for me*, *seeing the patients and seeing them decline and seeing them through the toughest parts*, *is definitely*, *like*, *I feel some relief when they finally do pass away*. (Clinical Team Focus Group)

Another salient message expressed by the clinical team regarding the death and dying process harkened on normalizing it:

*I do wish that there were more*, *enough studies*, *or just things in general that normalized the dying process*. (Clinical Team Focus Group)

Throughout the clinical team focus group discussion, staff members shared their observations of Last Gift study participants’ feelings and experiences at the EOL that informed how staff members viewed other aspects of the study, such as its potential for scientific advancement.

#### Perceptions about science and research

The clinical and rapid autopsy teams viewed the Last Gift study as an ennobling endeavor, and one that will further HIV cure science. Most notably, staff members highlighted the study as providing an opportunity for terminally ill individuals to be enrolled in research as well as serving as pioneers in engaging closely with the community from study outset. Additionally, staff expressed pride in the Last Gift study and that involvement in such an undertaking was “life changing”:

*[I] think of the Last Gift as a pioneer*, *as an example of how every study should be set up…with involving the community and listening to the next-of-kin*. (Clinical Team Focus Group)

*To be part of such an innovative endeavor is life changing*. (Rapid Autopsy Team Focus Group)

*I’m proud we pioneered it [the study]*. *I love the response from the community*. *Again*, *it makes me so proud every time that [person’s name] cites the Last Gift study as the example of the study that has had the perfect connection to the community*. (Rapid Autopsy Team Focus Group)

*To me*, *it’s…scientifically […] it’s going to be great*. *I mean*, *because now we could actually…we’ve been able to help people*, *you know*, *stay healthy because of antiretrovirals*, *but the one thing we haven’t been able to do really is to find where the latency reservoir is*, *and I think it’s just going to be exciting to be able to do that*. (Rapid Autopsy Team Focus Group)

*A lot of older people weren’t able to participate in research for many reasons*, *and they felt excluded from the whole research enterprise that they were so invested in at the beginning and have benefited so much from and people keep saying this is really an opportunity to give back again*, *and to get reengaged […] and improve the world*. (Clinical Team Focus Group)

The notion that the Last Gift study served as an ennobling endeavor and as a pillar for close collaboration with the community was evident on the part of the participants as well as the study’s staff members.

#### Perceptions about Last Gift participants’ interactions with study team

The clinical team described two key interactions: 1) relationship-building between the Last Gift participants and the clinical team, and 2) the team forming lasting connections with the participants’ next-of-kin/loved ones.

Relationships between the participants and clinical team expressed the trust, bond, and social support between parties:

*It has really been inspiring to see how much [Last Gift participants] have bonded with the team*, *how much being a part of this program*, *not just this study*, *has meant to them*. (Clinical Team Focus Group)

*[I] went to see [a participant] in the nursing facility where he […] really didn’t like the facility*, *and his face lit up absolutely*. *I mean*, *I walked into the room and he just changed*, *I mean*, *was happier*, *and it*, *I mean*, *it’s an honor to see that and feel that*, *um*, *I don’t get that very often from my normal patients*, *um*, *but it was*, *you know*, *it was a bond that I don’t know how to otherwise describe*. (Clinical Team Focus Group)

*I’m familiar with having many participants die who were living with HIV*, *and*, *um*, *one of the things that I often notice is that a few*, *quite a few*, *would die in isolation*. *They were older*, *end-stage organ disease*, *cancer*, *and something that I would do if I was there close to their time of death [is] I would call a significant other or a friend and just ask that person to speak to that person that was dying on the phone*, *and just put my cell phone next to their ear so that they could say their goodbyes*, *um*, *to the participant [and] that is something that I thought was very*, *very important*. *I’ve seen many individuals go from a very rigid body to a more flaccid*, *relaxed physical presence when they hear the voices of their children*, *of their parents*, *or of their significant others on the phone that can’t be there with them when they’re passing so…that’s something I try to do when I’m allowed to be present at the time of death*. (Clinical Team Focus Group)

Relationships between participants’ next-of-kin/loved ones and the clinical team highlight the psychosocial benefits of receiving support from the study team, particularly as next-of-kin/loved ones journey through the Last Gift participants’ death and dying process:

*[T]he next-of-kin that called us family to them made me really realize how much […] all the work that we did in advance that it’s almost more important to them that…that we go and regularly visit them*, *and I think that there is a psychosocial benefit that goes beyond being part of the research study just to have all of these extra people that care about you*, *and that visit you*, *and everything that we do for them*. (Clinical Team Focus Group)

*I personally check in with individuals and send them a text message and say that ‘I’m just letting you know that I’m thinking about you and if there’s anything that*, *any questions*, *or if you want to contact anybody on the Last Gift team*, *just give us a call*, *or send us a text message*,*’ so*, *I think that’s really important*, *um*, *the fact that we have next-of-kin or significant others participating*, *um*, *is extremely important in this study*. (Clinical Team Focus Group)

It is important to note that a few next-of-kin/loved ones shared feelings of discontent about the study:

*One thing that is very stressful to me in general with the Last Gift is when the next-of-kin are not happy*. *I feel that happened a couple of times right now…there was some disconnection about how much the Last Gift patients [participants] care about being in the study*, *and how when the next-of-kin feels we are a little bit intrusive*, *and I’m very stressed about it*. (Clinical Team Focus Group)

Clinical staff members noted discrepancies between the comfort level described by Last Gift participants compared to what next-of-kin/loved ones articulated with regards to being part of HIV cure-related research at the end-of-life.

### Perceptions of staff members’ close involvement in the Last Gift study

Five sub-themes emerged as the clinical and rapid autopsy teams described their close involvement in the Last Gift study: 1) cognitive processing, 2) resilience and managing stress, 3) coping mechanisms, 4) self-actualization and fulfillment among staff members, and 5) gratitude towards participants and the study.

Clinical and rapid autopsy team members articulated ways in which they processed their interaction with the Last Gift study, highlighting prominent emotions, their attachment to the study and its participants, and how the study shaped their worldview:

*[E]ach one of [the participants]*, *I remember every conversation we had*, *I remember what they like (…) and I remember every detail of every one of them*. *So somehow it is very special*. *Almost too much*. *I almost worry that sometimes I am almost a little bit too involved*. (Clinical Team Focus Group)

*[I]t’s pride in the people who came up with the idea and made it happen*, *because there wasn’t [chuckling] an easy list as you know in the beginning against you know to pull it all together and deal with some really negative reactions initially*, *so the fact that…we’re committed to what they did*, *reset the community the way they did*, *incorporated the social sciences the way they are*, *is a testament to the people involved*, *as well as the participants themselves of course*, *who have been waiting for something like this for a long time*. (Clinical Team Focus Group)

*[A] mixed bag of emotions*, *uh*, *trying to separate*, *like*, *the person and the actual autopsy and their stories and their lives that they’ve held*, *um*, *from like the science of it*. *And I don’t think that necessarily has to happen*, *and this study kind of brings it all together*. (Rapid Autopsy Team Focus Group)

Rapid autopsy team members particularly shared feelings related to coping with stress and building self-resilience:

*[I]t’s stressful I think when we know that the person is close to death because…I feel like if I’m at home*, *anytime I hear my phone ring*, *or like*, *vibrate or something I think*, *‘This is it*, *it’s the autopsy*.*’ I’m like already ready to drive over…but it’ll be someone else asking something but I feel like there’s…just always*, *‘this is it*, *this is it’ every time you get*, *like*, *any kind of phone notification*. (Rapid Autopsy Team Focus Group)

*[T]he only stress is just kind of*, *you know*, *people don’t die on a schedule usually…so there’s always a little bit of stress associated with being on call for anything…you’re working kind of strange hours*, *and you’re getting home at strange hours and that can be a little bit stressful*. (Rapid Autopsy Team Focus Group)

*I don’t really have stress (…) because I know we cannot control when they [participants] pass away*, *so I’m just like everyone*, *waiting and then*, *when we are there*, *we’re going to do the best we can*. (Rapid Autopsy Team Focus Group)

The rapid autopsy team reported conducting a “minute of silence” prior to performing each rapid autopsy, which alleviates stress and honors the participant’s lives and their contributions to science:

*[T]he minute of silence is huge […] before the autopsy…it says*, *‘From our first breath to our last*, *each of us tells a unique story with our lives*. *Today we honor our donor*, *[Last Gift Number]*, *for this opportunity to further research and to HIV and so many unanswered questions about the human condition*. *May we take a moment of silence now to honor his or her gift*, *and express our gratitude for all the discoveries this altruistic act will yield*. (Rapid Autopsy Team Focus Group)

The “celebration of life” event in honor of Last Gift participants after each rapid autopsy was another method reported by the rapid autopsy team that specifically helped team members cope:

*I really feel at the end of the autopsy I feel like I run five marathons*. *And the [celebration of life] at the end*, *yeah*, *that’s pretty important*. *Like*, *I know it’s important to many*. *It’s very important to me*. *(…) I think is a little bit of our way to cope with [the autopsy]*, *and I go home and sleep much better*. *I think the first couple of autopsies*, *we didn’t have the [celebration of life] after and I was going home with adrenaline [through] the roof*. (Rapid Autopsy Team Focus Group)

In tandem to resilience, stress, the minute of silence, and the celebration of life, both clinical and rapid autopsy teams expressed alternative coping mechanisms for approaching their involvement in the Last Gift study. Most notably, these mechanisms involved staff compartmentalizing emotions, performing physical relaxation techniques and exercises, separating their personal lives from professional, and “sublimating [their] burden into [the participants’] desire”:

*I’ve worked ICU [intensive care unit]*, *I’ve worked ER [emergency room]*, *and I’ve dealt with people dying constantly and I think as a result*, *I’ve learned to just not be [the participant’s] friend*. *I’m their clinician*, *and I’m there to do a job*, *and like I know that’s very robotic but it’s kind of my way of like trying to protect myself*. *The exception has been with one of the participants here*, *is someone I knew*, *and he continually was reaching out getting me to be more involved in his life*, *like*, *towards the end*, *and that was extremely difficult…but other than that I just*, *how do I deal with it*? *I took days off*. (Clinical Team Focus Group)

*[M]y approach of seeing all the things we’re doing as*, *um*, *exercising the will of the participant so I think that sort of sublimating my burden into their desire and it helps me cope with that*. (Clinical Team Focus Group)

*[Y]ou compartmentalize your interactions with dying individuals as job*, *and then try to keep personal separate*. *I think that’s appropriate*. *You’re going to be facing times when it’s not going to be possible to keep them separate*, *um*, *but exercising the muscle has to help*, *so I think that’s a good one*. (Clinical Team Focus Group)

*I’m a person of faith*, *so I do pray before I start my day and I have a yoga mat in my office and I do stretching in my office*. *I close the door and I put on my little “Do Not Disturb” sign out*, *and I do stretching exercises on the floor and I also do chair yoga*, *and then I pray at the end of the day*. *That’s how I deal with my stress*. *That and having a wife that cooks wonderful*, *too*. (Clinical Team Focus Group)

*I’m pretty good at compartmentalizing things so I think that’s how I handle stress*. *So*, *when I first walk in*, *I see the body*, *I’m sad*, *there’s grief*, *but with our minute of silence*, *or without sometimes*, *I give it that moment and after that*, *I’m game on*, *my head is in a different place and I just completely block everything out*. (Rapid Autopsy Team Focus Group)

The clinical and rapid autopsy teams articulated aspects of self-actualization and fulfillment related to Last Gift study involvement:

*I am a better researcher and a better person because of the Last Gift*. (Clinical Team Focus Group)

*I have been waiting for this study for three and a half decades and I really appreciate being here now on behalf of all my friends and my patients who aren’t here today*. (Clinical Team Focus Group)

*This study has definitely changed my life*. *I never imagined*, *I mean*, *that I would be part of such a study*. *I spent*, *you know*, *almost thirty years just doing regular scientific work and now I still am*, *but now I’m actually*, *uh*, *involved in this study that’s just so amazing*. *Somebody just giving themselves away to try to help others […] It has changed my life*. *And I*, *I think*, *in a way*, *it has made me a better person*, *because it’s made me more aware of*, *you know*, *like my team around me*. *It’s…it’s brought us closer*, *it definitely just lets me think about everything I do now and how it’ll impact someone’s future*, *and hopefully in a…in a good way*. (Rapid Autopsy Team Focus Group)

Gratitude was also a salient theme common among both clinical and rapid research teams as a result of involvement in the Last Gift study:

*I feel privileged…these [participants]*, *they’re dying and they’re letting us join their life…build relationship with these people as they’re going through one of the toughest things in their life*. (Clinical Team Focus Group)

*[A]s a young scientist*, *I am humbled to be a part of the Last Gift study*. *Uh*, *I think this is the most intimate encounter with translational medicine that I’ve encountered before*. *Um*, *and*, *it’s a good interaction for humanity and research*, *and ethics*, *and community*, *so I really appreciate this opportunity and I’m thankful for working with great leaders*, *and*, *uh*, *altruistic community*. (Rapid Autopsy Team Focus Group)

*[The rapid autopsy process] has made me a lot more appreciative of my research*, *because…that’s when you’re really connecting your work to what’s sort of impact it’s having…when you’re actually seeing a person there on the table and you’re thinking about their lives and […] how their lives may have changed if we had the answers that we’re seeking from their generous gift*. (Rapid Autopsy Team Focus Group)

Clinical and rapid autopsy team members expressed pride and privilege in being part of the study team, alongside feelings of gratitude and fulfillment.

### Considerations for successful and sustainable study implementation

Two key themes surfaced from study teams reporting on the nuances and considerations related to the Last Gift study implementation: ethical awareness as well as the need for sustained community and patient engagement.

Regarding ethics, the clinical and rapid autopsy teams raised the issue of ethics in relation to vulnerability of participants at the EOL as well as the need for respectful interactions:

*I didn’t find the participants particularly vulnerable*. *I felt that they knew what they were getting into … Um*, *and I think just but definitely his significant others definitely knew what was going on so*, *that part I felt good about*. (Clinical Team Focus Group)

*I definitely think there are ethical issues in a sense that we should always be concerned about the ethics of anything we’re doing and we tend to address them*, *and so I don’t feel that anything is an outstanding issue that is not being addressed*. (Clinical Team Focus Group)

*I don’t think there’s any ethical issues*, *and we’re all very respectful…When we later on take the tissues out of the freezer and we’re cutting*, *even at that moment*, *we’re all very respectful*. *We all remember what this is all about so I don’t think we have any issues with that*. (Rapid Autopsy Team Focus Group)

The study teams also noted the importance of managing study participants and their next-of-kin/loved ones’ expectations, underscoring the necessity of transparency and a well-informed consent process:

*For me*, *crucial is informed consent…we need to be very clear about what we are doing*, *the risk that people are going into*, *we need to make sure they understand it*, *the next-of-kin understand it…I do not think that our patients are vulnerable*. *I do think that our patients are empowered by having the opportunity to be part of this study*. (Rapid Autopsy Team Focus Group)

The clinical team particularly highlighted the significance of community engagement in study start-up and implementation, as well as how the study itself shifted perspectives for team members:

*I don’t think this program would happen at all if we didn’t have a strong CAB [Community Advisory Board] and strong community buy-in and investment*, *and I don’t see how this would ever work without that*, *and that takes relationships and it requires two-way communication and respect*, *um*, *that’s important*. (Clinical Team Focus Group)

*It’s really a textbook example of how you engage community in the development of research so kudos to [the researchers]*. (Clinical Team Focus Group)

The study’s processes are considered important elements of ethics as well as meaningful community and patient engagement, as described further below.

### Clinical and rapid autopsy organizational processes

While reporting on clinical and rapid autopsy processes, the study team emphasized three sub-themes: 1) the importance of learning about study implementation as an iterative process due to the novelty of the research, 2) nuances of engaging with various procedures, and 3) strong interdependence and team work between team members.

Regarding iterative learning, study teams emphasized different aspects of clinical and rapid autopsy processes that could undergo improvements with each execution:

*[E]mphasizing the learning process*, *being with the first participant and significant other*, *next-of-kin*, *um*, *I could tell that when the second one came*, *when the third one came*, *that there were things that changed just a little bit*, *slightly*, *I think to improve*, *um*, *the communication and the contact and the explanation of the study*, *because there were…I could tell that there was a lot of expectation in the beginning*, *just saying it’s an observational study is not enough*. *I needed to explain what that means and what it does not mean*, *and so*, *um*, *being more specific with each family as they came I think really helped out for the last few (…) donation autopsies that we’ve had to help the families prepare better for what to expect at the time of death*. (Clinical Team Focus Group)

*[F]or a couple of the participants and next-of-kin*, *they weren’t aware how ill their spouse was*, *which I thought was very interesting*. *So that was the learning process for me*, *and then that’s when I realized I really needed to have both the participant and the next-of-kin or significant other in the room at the same time so I could explain the study*, *because I knew that the participant was not transferring all the information to the next-of-kin or the spouse*, *and a lot of that had to do with [how] one was protecting the emotional stability of the other*. (Clinical Team Focus Group)

*I feel like we’re*, *we’re always learning*, *because we learn from like a previous mistake from the last autopsy and then we know that there is always something that we can improve in the next time*, *and we do that and*, *you know*, *we just keep*, *you know*, *we’re learning as we do each autopsy*, *so I feel like they’re all a bit different*. (Rapid Autopsy Team Focus Group)

Be it contact with Last Gift participants and their next-of-kin/loved ones or rapid autopsy procedures that required course corrections, the study team approached these nuanced processes with context and a responsibility to honor the participants’ wishes:

*I said to myself at one point*, *like*, *‘is this too much*? *Am I doing too much or interfering too much*?*’ And I rephrased it in my head*, *I said*, *‘I was there when he was consented*, *he truly wants to be part of this study*, *he wants this outcome*, *the outcome being successful rapid autopsy*.*’ So*, *when I do any of these things…I am doing it for his wish*. *And I think that same instance*, *that same emotion*, *when you hear that they passed away*, *this is… [It] is now my responsibility to fulfill that wish that he expressed to be part of the study and have the best outcome possible*. (Clinical Team Focus Group)

*I can’t tell you which is more important–the contact I had with the next-of-kin*, *significant other*, *or the contact that I had with the pathology team*, *but they are equally have very*, *very important outcomes*. (Clinical Team Focus Group)

*[I]n the autopsy itself*, *even as much as I prepare every single time*, *something happens that we don’t expect [chuckling]*. *It’s…it’s really just organized chaos*, *but it works in the end*. *I prepare seven hundred plus tubes every time*. *I label them all*. *I prepare the media*. *I have lists of what to do*. (Rapid Autopsy Team Focus Group)

*From a very young age*, *I was exposed to dead bodies*. *Open caskets*, *wakes that lasted days with a dead body just there* …*to me*, *there’s no fear*. *Uh*, *for the Last Gift though*, *I’m just in awe*, *there’s sadness because I know this person gave up something*, *but…but otherwise I’m okay with it*. (Rapid Autopsy Team Focus Group)

Strong interdependence and team work between Last Gift study clinical and rapid autopsy team members were made evident by the following quotes:

*The Last Gift has been a family*. (Clinical Team Focus Group)

*[I]t’s those times when you’re feeling a little too much of a tug from a participant you know […] we’re able to sort of lean on each other for things…whatever is needed*, *we’re there for each other to fill in a spot where being alone in that role would be difficult*. (Clinical Team Focus Group)

*I’m…in awe of how the team works together*, *um*, *because I really don’t think I’ve ever been part of a group that was so coordinated*. *It’s really impressive and I’m especially impressed by the leadership of the team too…So*, *you know*, *it means a lot to me on so many different levels to see the way the team has integrated and also*, *um*, *just to think about why we are there and the trust that people have put in us to be there*. (Rapid Autopsy Team Focus Group)

Similar themes from Last Gift study processes and implementation were salient in the study team’s portrayal of potentially expanding and scaling up the study to different clinical research contexts and settings due to the community’s willingness to participate.

### Considerations for study scale-up at other clinical research sites

The clinical and rapid autopsy teams articulated the Last Gift study’s potential replication as one where ethical community engagement principles and strategies, an established and interconnected study team, and strong relationships between the study team and participants (and their next-of-kin) would be of necessity:

*[T]he team is a little bit special here*, *and I’m not sure we can reproduce it so easily*, *honestly [chuckling]*. *And the community…I think you could not do something like [this study] without the community buy-in*. (Clinical Team Focus Group)

*[H]ow you interact with the patients and the families*, *which is an amazing skillset that you can’t necessarily teach and not easy to find*, *and then engaging the community and getting that proper support so you don’t get in any*, *uh*, *um*, *unanticipated blowback*. (Clinical Team Focus Group)

*[W]e have an amazing team and I don’t know if anyone is ever going to be able to duplicate it*. *Um*, *we*, *a lot of us have worked together for a few years*, *and*, *and even in the laboratory*, *[…] we work so well*, *it’s like a well-oiled machine*. *And I’m glad that all that has transferred into the rapid autopsy room*, *um*, *because of so many things going on*, *and the stress level*, *it’s just*, *it just works*. *The team just works*, *and to me*, *it’s a real privilege to work with such wonderful people and I would do it all over again if I were asked to*. (Rapid Autopsy Team Focus Group)

Clinical and rapid autopsy teams feel it might be difficult to fully scale-up the Last Gift study’s processes and framework because of the strength of established relationships between: 1) the study team and HIV community, 2) study team and the participants/their next-of-kin, and 3) study team members themselves. The Last Gift team also felt the necessity to ensure the sustainability of the program through self-reflection and team care.

## Discussion

Focus group discussions with the Last Gift study’s clinical and rapid autopsy team members revealed perceptions and experiences that team members had with the study’s participants and their next-of-kin/loved ones, the community, and the study’s overall implementation, processes, and considerations for future scale-up at other research sites. To our knowledge, this is a rare account of perceptions and experiences of research staff members involved in HIV cure-related research. Clinical and rapid autopsy staff members revealed deep gratitude for and recognition of Last Gift study participants’ altruism, fulfillment, and control at the EOL, further expressing participants’ peace and acceptance in the death and dying process as a result of study involvement. Staff members shared the continual learning and staff member interdependence that take place in all clinical and rapid autopsy processes, also emphasizing various coping mechanisms, stress management techniques, and importance of integrating ethics [1] as well as community and patient engagement. Understanding these complex emotional and structural networks that affect the execution of intensive HIV cure-related research at the EOL is crucial to such research studies’ sustainability, which the socio-behavioral sciences literature currently lack.

Clinical and rapid autopsy staff members shared similar perceptions about the Last Gift study, citing participants’ altruistic motivations, empowerment, and control at the EOL. These accounts are consistent with the emerging socio-behavioral sciences literature on HIV cure-related research, emphasizing altruism as a primary motivator to participation [2, 4, 10–17]. Clinical and rapid research autopsy staff also related these insights to the study’s broader scientific significance and its pioneer status. Similar altruistic motivations and a “gifting relationship” were echoed by terminal cancer patients who participated in rapid tissue donation programs [18].

Interdependence among staff members was a crucial pillar to sustainable study implementation–not only for the execution of specific tasks (i.e. rapid autopsy procedures) but also provision of social and emotional support for team members, study participants and next-of-kin/loved ones. These themes were intermixed with staff members’ self-determined responsibility to honor the Last Gift participants’ wishes with dignity and respect as well as successfully accomplish their respective tasks. This articulation of personal responsibility is a hallmark of the “provision of high-quality care” [19] at the EOL, as echoed in existing literature that it is of utmost importance to honor patients’ wishes and hold dignity and ethics at the forefront of EOL work, which can be emotionally charged in itself [1, 20–24]. Additionally, as EOL and organ donation ethics are evolving and may be subjected to contention [25], the study team must ensure patient autonomy [22], address challenges swiftly, and course correct as necessary [26]. Such practices are also true within the field of HIV cure-related research field at large given the innovative and challenging nature of the research [27].

From their depictions of study-related experiences, clinical team members appeared to exhibit acts of kindness, respect, and care that showed they were cognizant of potential pain and isolation for PLWHIV at the EOL, while balancing the reality that the Last Gift is a research study and does not involve palliative care [28–30]. Notably, the Last Gift study research coordinator has over 30 years of experience in HIV care and participant-facing aspects of HIV research, harboring important skills that allow the coordinator to interact with participants at the end-of-life as well as next-of-kin/loved ones with sensitivity, patience, and authenticity. Rapid autopsy team members exhibited similar characteristics, honoring the participant’s premortem decisions, respecting their “gift” with a minute of silence before each rapid research autopsy, and treating the body with respect throughout the entire process [6, 20, 31–34].

The overall process of death and dying can be potentially riddled with lack of comfort and closure at the EOL [30]. The Last Gift study team reported that for study participants, being involved in the study provided them with a layer of peace and control that they would have otherwise not had. Such peace, coupled with an established care relationship with the study team, helped ease the death and dying process for participants. Similar sentiments were echoed in existing literature regarding a strong participant-study team care relationship (or strong patient-physician relationship [35] as central to ethical support without intrusion on family care structures [20]). The Last Gift study team capitalized on patient/participant-centeredness through their continual emphasis on adhering to participants’ wishes, establishing trust in the participant-study team relationship [1], and incorporating participants’ perspectives into the learning process and research continuum [5]. It is important to note that a few discrepancies were noted between the Last Gift participants’ comfort with the study compared to their next-of-kin/loved ones’ comfort. Additionally, one clinical team member noted that there were divergences between the participants’ experiences of illness compared to their loved ones’ perceptions of the participants’ illness, hypothesizing that this difference could be due to participants’ attempts to shield their loved ones from negative feelings.

Interestingly, some Last Gift study team members reported having difficulty separating the personal from professional with regard to feeling connected to participants and coming to terms with their impending death, reporting attempting to compartmentalize their feelings, “sublimating” their burden into the participant’s “desire” to contribute to the study, and taking time off from work, among other coping mechanisms. In tandem, staff members expressed gratitude, self-actualization, and fulfillment as a result of Last Gift study involvement, in addition to different emotional and cognitive processing methods and compartmentalization techniques to cope with the sensitive attributes of this EOL HIV cure-related research study. Particularly for rapid autopsy team members, the Last Gift study seemed to be a constant presence in their lives (i.e. at home, outside of work hours, etc.), suggesting that these team members, and to a lesser extent, clinical team members, should be equipped with resilience and appropriate coping mechanisms to handle stress and emotional processing in a healthy manner. Existing literature on staff members’ experiences and perceptions in EOL HIV cure and rapid autopsy research is limited, though one study described physicians’ experiences in EOL HIV care and reported similar psychological challenges related to the physician-patient connection and patient empowerment [36].

Staff members’ engagement with the HIV community and the Last Gift participants’ next-of-kin/loved ones was also described as an invaluable driver toward successful and sustainable study implementation. These findings are consistent with existing EOL research with end-stage cancer patients that involved family members in the consent and funeral arrangement processes [20]. Staff members strongly believed in the strength of established community networks and interconnected research infrastructure that allowed for study start-up and implementation. Thus, some staff members expressed skepticism that the Last Gift study could be completely scaled up or reproduced in another setting and articulated that thorough guidance documents and procedures should be outlined to undertake EOL HIV cure-related research. Research groups and institutions planning to implement similar EOL HIV cure research projects could visit current research sites to engage experienced staff and discuss lessons learned. Current EOL HIV cure research sites could also host workshops and create research collaboratory to provide a mechanism for effective replication, collaboration and scale-up. Currently, similar EOL HIV cure research projects are emerging in the United States, South Africa, Canada [4, 5] and Europe. It would be interesting to collect staff perspectives in more diverse settings to enrich the literature.

We must acknowledge a number of limitations for this small focus group study. These include the conduct of only two focus groups at a single clinical research site, the potential for social desirability bias and ‘group think,’ and the lack of generalizability. Quotes were not ascribed to staff participants due to difficulty in identifying participant voices from audio files and the general nature of focus groups. Nevertheless, given the very limited prior research on staff perceptions and experiences in conducting HIV cure research at the EOL, we believe our findings pave the way for the prioritization of staff members’ concerns and needs in EOL HIV cure-related research.

**Table 3**summarizes our key findings and possible implications for EOL HIV cure research.

**Table 3:** Summary of findings from focus group discussions with Last Gift clinical and rapid autopsy team members and possible implications for EOL HIV cure research.

Summary of Findings	Implications for Future EOL HIV Cure-Related Research
**Perceptions of the Last Gift Study**	
• Clinical and rapid autopsy team members described Last Gift participants’ feelings of altruism, fulfillment, empowerment and control at the EOL, and the death and dying process as a result of study involvement.	• Clinical and rapid autopsy team members’ interactions with study participants and their conceptualization/understanding of the study’s overarching mission and aims can shape the study’s execution and trajectory.
• The study team expressed that trust, bonding, and care were central to their relationships with participants, providing their next-of-kin/loved ones with psychosocial support during the death and dying process.	
	• Prioritizing patient/participant-centeredness and trusted care relationships can provide solace and control to study participants, easing them through the death and dying process, as well as provide the participants’ next-of-kin/loved ones with unexpected support and care, even though there must remain a clear distinction between research and clinical care, and a clear explanation that the study does not involve palliative care.
• Study team members described the study as a pioneer for scientific advancement in EOL HIV cure-related research.	
**Perceptions of Involvement in the Last Gift Study**	
• Clinical and rapid autopsy team members expressed gratitude, fulfillment and self-actualization as a result of study involvement and shared experiences related to resilience, stress management, and coping methods pertaining to the study’s sensitive features (i.e. performing a rapid autopsy and providing compassion to participants and next-of-kin/loved ones at the EOL).	• Understanding how clinical and rapid autopsy team members react to EOL HIV cure research studies’ sensitive attributes is crucial to equipping members with adequate support structures to preserve their mental health and ensure study rigor and sustainability.
**Last Gift Study Implementation**	
• Ethics as well as community and patient engagement were found to be critical elements to ensure successful implementation of EOL HIV cure-related research.	• EOL HIV cure-related research studies cannot be executed without a strong, sustainable partnership with the community and adherence to robust ethical practices adapted to the EOL and study contexts [1].
**Clinical and Rapid Autopsy Processes**	
• Clinical and rapid autopsy team members emphasized learning as an iterative process throughout study execution and underscored the importance of interdependence and team work between staff members.	• Interdependence and team work between study team members were critical features of EOL HIV cure-related research that should be prioritized, as some roles/procedures may be physically and emotionally taxing.
	• Rapid research autopsy studies may require professional and social support.
**Potential for Study Scale-Up and Sustainability**	
• An established and interconnected study team, ethical community and patient engagement, and strong relationships between study staff and participants (and their next-of-kin/loved ones) will be necessary characteristics for scaling-up EOL HIV cure-related research at other clinical research sites and ensuring sustainability of these program.	• In order for the Last Gift study (and other EOL HIV cure-related research studies) to be scalable and reproducible, recommended procedures should be created to ensure community engagement, relationship-building between study staff and participants, and a strong, coordinated team, taking into account different sociocultural contexts.
	• Potential mechanisms to disseminate information or ensure effective collaborations related to EOL HIV cure research include 1) workshops hosted by current EOL HIV cure research sites, 2) on-site visits by research groups interested in implementing similar research, and 3) establishment of a research collaboratory mechanism.

## Conclusions

Understanding the complex procedural and emotional nuances involved in conducting HIV cure-related research at the EOL is crucial to the Last Gift study’s success and sustainability. Exploration of the study’s potential scale-up or reproducibility in other contexts will require an established research infrastructure with supported clinical and rapid autopsy teams and trusted community buy-in channels for ethical engagement with PLWHIV and affected communities.

## Supporting information

S1 Appendix Contains supplemental quotes from the Last Gift study’s clinical and rapid autopsy staff members that were not included in the Results section’s main body. (DOCX)
