# Supplementary material for: “[It] is now my responsibility to fulfill that wish:” Clinical and rapid autopsy staff members’ experiences and perceptions of HIV reservoir research at the end of life
Source: PLoS One. 2020 Nov 18;15(11):e0242420. doi: 10.1371/journal.pone.0242420 (PMC7673534; doi:10.1371/journal.pone.0242420)
Supplement: S1 Appendix — (DOCX) [file pone.0242420.s001.docx]

**S1 Appendix: Supplementary Quotes**

| **Themes** | **Quotes** | **Type**  (Clinical vs. Rapid Autopsy Team) |
| --- | --- | --- |
| **Perceptions of the Last Gift Study** | | |
| **About Last Gift Study Participants** | | |
| **Altruism (as demonstrated by Last Gift participants)** | | |
|  | *This is a population that has lived as long as they did because of advances in research. And I think especially in the HIV community, people are acutely aware of this and many people were involved in the research as participants, they literally put their bodies on the line at the beginning of the epidemic and now that they are, um, you know, facing their own deaths, you know, and it’s different for this population that people were thinking…I remember some of the questions at the beginning were whether or not people would want this to happen to their death and if they wanted HIV to be part of their death and they were just adding too much to people.* | Clinical Team |
|  | *[T]he Last Gift means a lot to me. Having been in HIV research so long, I think that it is, um, wonderful, and so unselfish of the participants to, uh, for their very last act to donate their bodies so that we can make a study.* | Rapid Autopsy Team |
|  | *I just think for the participants, they just feel like their leaving their last mark and they know that their death is going to help others.* | Rapid Autopsy Team |
| **Fulfillment (as expressed by Last Gift participants)** | | |
|  | *[I]n the moment of life where it feels like [the participant] is losing everything, being a part of a study like the Last Gift, where you feel the legacy that you can leave, you feel you’re a part of something bigger, and also how the Last Gift has been implemented in a way that makes your life significant and important, I think it can really made a difference.* | Clinical Team |
| **Empowerment & Control at the End of Life (as demonstrated by Last Gift participants)** | | |
|  | *…at the end of life there is control over HIV a little bit because of the study and maybe that gives them solace as well.* | Clinical Team |
|  | *I push back against [the term vulnerability] because I don’t think that we should be treating people that way, that they are empowered, and able to make their decisions.* | Clinical Team |
| **Peace in the Death & Dying Process** | | |
|  | *…it has been really…meaningful to watch how much…meaning and dignity this study seems to have brought to people who are facing their own death.* | Clinical Team |
|  | *I think a lot of the participants did die peacefully…this [study] brings a lot of value and makes the process much easier.* | Clinical Team |
|  | *I think we’re in a society that doesn’t handle death as well as we used to a hundred years ago and, um, when I described the study to lay and medical people, they cringe, as the most common reaction, and particularly sort of the idea of doing the rapid autopsy, I think, I guess puts people off, but I think emotionally, that’s probably, yes, what was hard to get over at first, but seeing people enroll and seeing how they engage with the study and are pleased to be in it, and have that control, and have that contentment, um, makes me want to keep doing it.* | Clinical Team |
|  | *When you have the next-of-kin involved for research purposes, it’s been my experience that you just have a better outcome, and I’ve seen that in the Last Gift. It’s, um, it really improves the outcome and I’m glad that we have it structured the way it is because I don’t think it would function any other way, and I also think that it helps the participant cope with their, um, death, and their pain.* | Clinical Team |
|  | *From a medical standpoint, um, to meet death is a, I mean, I hate to say this, it’s kind of a medical procedure, it happens, you know. We proceduralize it all the time in our jobs, and this takes it away from a procedural event and makes it much more personal. Um, and these deaths, the ones that I’ve been involved in, which are only a small number, become much more personal and much more family-like, um, and, um, you know, the one that I was the most closely involved in was the very first participant, which left a very deep mark on me, and, um, it was very meaningful to be with him right at the very end, and, um, in particular, his partner didn’t know what to do and, you know, the participant was in a lot of pain, and…it was just, you know…I felt like I was his family being there. It was very nice. It was nice to be there to be able to help him.* | Clinical Team |
|  | *Our transportation team…oftentimes ask[s] the family, or the significant other or next-of-kin what kind of music did the now deceased like? And they actually have a variety of tapes and they play that music on the way to the morgue at UCSD.* | Clinical Team |
|  | *As the person who is sick, you spend a lot, [chuckling] invest a lot of energy, managing the emotions of people around you and dealing with their emotions, which may have a lot or nothing to do with you but yet you feel like you have to and it becomes really exhausting so…it would be interesting if we can see there are other ways to manage that but it’s something we have to keep in mind, but. The person who was ill, like they’re facing their own demise and they’re at peace with it, but the people around them may not be and that’s really taxing on the person themselves. (Clinical Team)* | Clinical Team |
| **About Science & Research** | | |
| **Last Gift Study as an Ennobling Endeavor, Pioneer, & Vessel for Scientific Advancement** | | |
|  | *[T]he Last Gift means a lot to me. It…definitely set the bar for any study very high, as you can get such a wonderful mix of extremely high scientific outcome, involvement with the community, ethics, um, and I see how enthusiastic the community is about the Last Gift, and being part of it since the very beginning and the complexity of all the parts that we have put together, and how the team has worked…it’s just at a completely different level than anything I’ve seen so far.* | Rapid Autopsy Team |
|  | *[W]hen you tread new territory like this that you are going to get everybody telling you, ‘Oh there’s going to be this wrong with it,’ and ‘This is going to happen,’…but I think it’s everything that individuals have said were, um, conflicts or ethically questionable that our participants and their significant others have in fact said totally the opposite, that they feel how important this study is and how supported they are and that next-of-kin appreciate that we continue having contact with them after the participant has died.* | Clinical Team |
|  | *We’re talking about this forward gift really down the road that’s happening, and, um, and I think, you know, it has to be sort of different. All of a sudden, the blood draws are not because your doctor is trying to figure out what’s going on with you but the blood draws are a part of the study, like something that you’re doing that still gives your life meaning. And to the point, I mean, [Last Gift] 8 at some point, started asking his clinical team to not draw blood because he didn’t want them to draw it as often, but he would still let us draw when we came. And I think that new, um, role that he had might have been a benefit.* | Clinical Team |
|  | *[A]s a member of the rapid autopsy team, I think it’s given me a greater insight into what kind of determination and, like, forethought it takes to complete this type of unique study, so I’m grateful to be a part of that innovative research.* | Rapid Autopsy Team |
|  | *We talk a lot about the ethics of being involved in the study and participants who are at end of life…the research to me is so impactful and has such great capacity for discovery that I really am excited for that.* | Clinical Team |
|  | *I’m excited from a scientific point of view, of course, because I know that [the study] is unique and we will learn so much, but everything that’s behind the Last Gift makes it even more special.* | Rapid Autopsy Team |
|  | *I feel like it’s a lot to decide that you’re going to donate yourself to science, but I feel like with this study, we’re going to be able to find out a lot of information. And I’m hoping that we’ll really be able to find a cure after going through all this information and doing all of these experiments.* | Rapid Autopsy Team |
|  | *I don’t always think about how kind of cutting edge it is, because, you know, it’s just my job for me, it’s what I’m doing, but I have to realize that, you know, if I don’t do a good job in the autopsy room, a lot of the downstream things are kind of meaningless, you know, if we don’t preserve something properly, or, you know, miss something, we just completely lose out on that, so it’s a little high stress in that sense, but it’s completely and totally worth it. I mean, it’s super cutting edge, and who really does tissue studies for HIV and rapid autopsies…it’s…I don’t know…it’s just a privilege to be a part of.* | Rapid Autopsy Team |
|  | *[T]he science is really just getting started, and hopefully we have some really good questions we can answer, and I think we can answer…those are still on their way, but even if we don’t have great earth-shattering findings, we still have the research participation that I think has, uh, really opened up my eyes and are research opportunities in this area.* | Rapid Autopsy Team |
| **About Last Gift Participants’ Interactions with Study Team** | | |
| **Relationship Between Participant and Study Team** | | |
|  | *[…]you see [the participants], check on them, you hear how they’re doing, their struggles, and when they’re doing better, when they’re doing worse, you know, so…it’s…like having been a nurse for as long as I have, I’ve never had that relationship with, like, primary care almost. This isn’t primary care, but… it has the same feeling I feel like, and it’s definitely a new twist for me.* | Clinical Team |
|  | *[T]here are a couple of participants that really don’t have people visiting them, or that they could reach out to or talk to in any capacity more than, you know, basic conversation, so I think those participants especially have a lot to benefit from this.* | Clinical Team |
|  | *[T]here was…some intermixing between my role and the clinical team… When I was seeing the patient, they were asking me questions all the time, and sometimes, I had an opinion about it, and sometimes, the opinion I had about it was not necessarily what was going on… So I find it, like, a difficult line to walk.* | Clinical Team |
| **Relationship Between Participants’ Next-of-Kin and Study Team** | | |
|  | *[I]f you only spoke to just the participant and really didn’t get a chance to speak to next-of-kin or significant other until after death, then they’re just…I think we’re missing out on something when we don’t involve them closely in the beginning.* | Clinical Team |
|  | *[I]t’s a study that’s not just for the participants but also for the next-of-kin and that’s a strength of the study but also something we have to keep in mind.* | Clinical Team |
| **Perceptions of Involvement in the Last Gift Study** | | |
| **Resilience & Managing Stress** | | |
|  | *I think the only stressful part is just waiting for them to pass and not knowing, like, specific timing and schedules.* | Rapid Autopsy Team |
|  | *I don’t feel stressed so much. I understand…my professional life being on call almost all the time, it…I think that I’m more used to it. But maybe we should…think of ways that we can more formulize back up and being able to have people really have time off and being on call might decrease some of that stress or at least understand some of the processes and…can, uh, relieve some of that stress.* | Rapid Autopsy Team |
| **Coping Mechanisms** | | |
|  | *[Name] and I were together at [conference] after LG05, and we were meeting people who were…working with the tissues from his autopsy and…I said, you know, ‘we were there the day he died, but right now he, parts of him are alive in this research, like literally cells were still alive.’…[H]e was somebody that, an illness took him way before his time, and he wanted to be a part of this study, and, and knowing that our work was letting him just evade the angel of death a little bit longer, I think, it made me happy.* | Clinical Team |
|  | *[Person 1] I feel like I have this deep emotional part but then this very pragmatic part in everything I do.*  *[Person 2] I think the psychiatrist would call that emotional compartmentalization…I think it’s one of the healthy coping mechanisms.* | Clinical Team |
|  | *I don’t…I don’t think in the autopsy although there’s so many things going on, but I don’t really feel stressed because I know that very good people have taken care of things that we need, that we know how to communicate, we know exactly what to do, we work well as a team, and we’re so aware of each other’s needs, uh, so to me personally, doing the autopsy, I…we’re busy, we’re rushed, but to me, it’s not stress. To me, it’s…we need to get this done, this is work, it’s not stressful.* | Rapid Autopsy Team |
|  | *I tend to get very emotional, so my heart is usually 120 beats per minute for like six hours…the decompression [hour] with [the celebration of life] with pizza and wine in the lab is, like, the one thing I look forward to [chuckling] as the way to process it, and the only way I can go to bed after the rapid autopsy.* | Rapid Autopsy Team |
| **Self-Actualization & Fulfillment** | | |
|  | *[The Last Gift] has made me a better person and researcher.* | Clinical Team |
|  | *I feel like my life is almost like a before and after the Last Gift, I feel, like, maybe because I’ve been part of it since the very beginning, my career has really been driven by the Last Gift.* | Clinical Team |
|  | *The Last Gift study…definitely changed my outlook on life.* | Rapid Autopsy Team |
| **Gratitude** | | |
|  | *As a person living with HIV, this is exactly the kind of research I’d always hoped there would be. That’s really gratifying to see that this has come to fruition and that I get to be a part of it as well.* | Clinical Team |
|  | *I definitely value...being a part of a study that is more…directly impacting my community, um, as a gay man.* | Clinical Team |
|  | *I really thank the researchers for coming up with this study because it means a lot to me personally as well as professionally.* | Clinical Team |
|  | *[I]t has been inspiring to me to see how rewarding this [study] has become for both the participants and the people on our side of the study.* | Clinical Team |
|  | *I’m proud of being a part of [this study].* | Clinical Team |
|  | *I take [this study] very seriously and I…I just think it’s really great that these participants and their families are willing to do this. And I think that it’s really great for the rest of the team to want to participate in such a wonderful thing scientifically and also on a personal level.* | Rapid Autopsy Team |
|  | *[I]t’s really important to me being, you know, just like a small part of [the study], because even though I can’t donate my entire body, I can donate just a little bit of my time to help.* | Rapid Autopsy Team |
|  | *[I]t’s an honor and a privilege to be in the rapid autopsy team. I mean, I was, um, I was honored that I was even asked to be on the team because I think it’s…very valuable part, not the most valuable part but it’s a very valuable part. I mean, that is, is when, you know, your job in that autopsy room is to make sure you preserve the tissue and, so that you can do all the downstream applications later on to try to answer all the scientific questions that have been laid out.* | Rapid Autopsy Team |
|  | *I am in awe of the fact that someone has put their trust in us and also me as a member of this team to do something that’s so important.* | Rapid Autopsy Team |
|  | *Every time I can explain to someone what the Last Gift is, I will do it, just because to me…I’m really proud to be part of it.* | Rapid Autopsy Team |
|  | *[W]hen I explain, kind of, how this is new in HIV research, that usually people don’t have those parts of the body available to them, [my friends and family] are usually pretty impressed. It’s also just really humbling that this person donated their body to us. That’s amazing, and not only that, since it’s rapid, they’ve donated possibly their family members’ time at the end where they don’t have as long to say goodbye.* | Rapid Autopsy Team |
|  | *I…feel honored to be a part of this study.* | Rapid Autopsy Team |
|  | *[I]t’s amazing to know that these people are…are giving so much of themselves to further research that could help, you know, millions. And so, um, I am really honored to be a part of that.* | Rapid Autopsy Team |
|  | *[T]o spend so much time thinking about what these people have given is life changing, and…it doesn’t change my day-to-day…but it does affect my outlook and my appreciation for my research.* | Rapid Autopsy Team |
|  | *I consider more the viewpoint from our study participants, because it’s a lot to give that kind of commitment at the end of life and I guess for me, I wonder that if I were in their shoes, if I would be able to give the same amount of time that they are and, you know, just as far as like, understanding like what I’m, I guess, maybe sacrificing in other aspects of my life towards the end of my life.* | Rapid Autopsy Team |
|  | *I do appreciate that we take a moment of silence before we start, um, the autopsy and I think that helps me refocus and truly appreciate everything that the community has given and…what we’re here for.* | Rapid Autopsy Team |
| **Considerations for Last Gift Study Implementation** | | |
| **Ethics** | | |
| **Ethics & Vulnerability** | | |
|  | *It’s really gratifying as a community research activist to see that this has been done right from the beginning. (Clinical Team)* | Clinical Team |
|  | *[M]y big ethics issue was, you know, the one I keep mentioning was the patient that I knew, so just kind of the…definitely the gap of being his friend as well as like trying to collect data and like be part of the study. So there was that element of it. Um, let’s see, I do think they’re vulnerable, um, I think that’s in my opinion, pretty clear, you know, how we are honest and open and clear with our intentions and what we were doing so that we’re not misleading them or taking advantage of their vulnerability.* | Clinical Team |
|  | *[A]s long as we’re all respectful, which we are…I will be extremely bothered if somebody was in the autopsy making any jokes about the patient, or anything that will make me feel that we’re not respecting the body, which is not the case…I think for the rapid autopsy part…I think we make it in such a respectful way with a minute of silence…I don’t have any, I think ethical concern.* | Rapid Autopsy Team |
| **Community Engagement** | | |
|  | *It’s really a textbook example of how you engage community in the development of research so kudos to [the researchers].* | Clinical Team |
|  | *[The] Last Gift [study] changed my perspective about the community.* | Clinical Team |
|  | *I think that we need to do something somehow to make it more, um…more known out in the community to a variety of communities that there is research available for, um…people from different, in different areas, of which organ donation is one, and is a significant and important one.* | Clinical Team |
| **Clinical & Rapid Autopsy Processes** | | |
| **Learning as an Iterative Process** | | |
|  | *I feel like we’re, we’re always learning, because we learn from like a previous mistake from the last autopsy and then we know that there is always something that we can improve in the next time, and we do that and, you know, we just keep, you know, we’re learning as we do each autopsy, so I feel like they’re all a bit different.* | Rapid Autopsy Team |
|  | *[H]aving to…put together the Last Gift from scratch, there’s a lot of stuff that I wished I would have known, because honestly…everything that we had to put together we had to learn and looking back right now, the amount of work that we put in there, all of us during the last couple of years has been gigantic…I’m so proud that we got here right now with the protocol that works mostly, and we are still improving it, and like, all the pieces fall together, and that we were able…to make the autopsy work for all of our participants without ever failing.* | Rapid Autopsy Team |
|  | *[T]hings that I would want to know before…there’s lots of things – how big to cut, how to get the process moving smoothly…but I think that’s just because we were the first ones to do it, so those things we had to learn on our own and hopefully we can teach the future by coming up with a good protocol that others can follow. So, you know, that’s the problem of being trailblazers. You gotta make the trial and know which way to go.* | Rapid Autopsy Team |
|  | *[A]s far as improving during the autopsy, I guess, that will always happen as we do more autopsies, because we’re always learning from the previous one. So, I think it’s just going to be like…a constant level of improving every time.* | Rapid Autopsy Team |
| **Nuances of Engaging with the Process** | | |
|  | *In the beginning, it’s all about the participant, but as soon as the participant passes away, then my contact and communication with the next-of-kin, um, kind of goes into high gear leading them through the death certificate process, um, through the cremation, through their grief, assisting them in whatever communications they need to make with others, and then keeping in contact with them for a while afterwards.* | Clinical Team |
|  | *I wanted to point out some of the, I guess, day-to-day changes I made in my life as a result of this. Like, for example, if I’m wearing heels when I go out, I’ll pack sneakers in the car in case I get called [background laughter]. I carry transport paperwork, parking permits, and, um, an updating sheet of what to do for collaborators in my purse wherever I go [chuckling]. Um, my phone is on loud when I sleep [chuckling], you know, just little things like that. Oh, and I definitely sometimes will get like a stress dream if I don’t have a set prepared for autopsy.* | Rapid Autopsy Team |
|  | *[A]s much as chaotic as it is, and as crowded as it is in that one, it is also to me autopsy is like a well-oiled machine. I mean, it’s amazing on, uh, how smoothly things go and how people know each of their functions. Uh, a…a lot of us switch functions all the time, because we’re kind of familiar with it.* | Rapid Autopsy Team |
|  | *Autopsy means ‘okay, this is where we need to do the right thing.’ This participant has donated their body. Let’s get these tissues, let’s preserve them, let’s preserve them correctly, let’s store them correctly, and let’s just do everything possible so their…their donations, their full-body donations, what their families sacrificed, it doesn’t go to waste, that their legacy is left behind and it’s going to be something good that can help others.* | Rapid Autopsy Team |
|  | *[Autopsy] is just trying to get through everything because it’s physically really taxing because you’re running around, you’re never sitting, it’s hot, it doesn’t smell great, but you just have to get through it, because you owe that to the participant.* | Rapid Autopsy Team |
|  | *[The process] was very organized. I knew exactly what I was doing and it went smoothly, so I appreciate that. I appreciate all of the list of to-do things, and like, how to prepare these reagents to properly store tissues. So, I think at that point, it was really a well-oiled machine.* | Rapid Autopsy Team |
|  | *I don’t get to see a lot of the preparation that goes on beforehand…it’s something that I’m aware of, the technician team does a tremendous amount of work to get tubes labeled […] and everything ready to go…but when we get called in, the first thing that I really see is there’s a person’s body on the table, and um, you know, there’s a pathologist there ready to autopsy that person and so, um, you know, as the process goes, we get organs and then…my role is to actually cut small pieces for the samples that we’re going to need for all of our molecular work…that’s when it comes in handy to have all of those pre-prepared tubes…that the team has worked so hard to prepare. Um, and then, there are other people down kind of the assembly line that do things like homogenize the tissues so that we can get access to the RNA and the DNA, um, and stuff like that.* | Rapid Autopsy Team |
|  | *I am not afraid of dead body…I was probably one of the few persons in the team that knows the participants before and after and that never really bothered me. But it’s strange, yes, it is surreal. When you stop and think about it…I tend to not stop and think about it, but when you stop and think about it, it’s just strange...* | Rapid Autopsy Team |
|  | *When we go do the autopsy, it’s like adrenaline is running, we’ve got things to do, trying to be organized and not scattered [chuckling] and keep the task and then after you’re in the decompression stage, I think is the time where there’s reflection and like, ‘wow, that just happened.’* | Rapid Autopsy Team |
| **Interdependence and Team Work (Between Staff Members)** | | |
|  | *[T]he biggest thing that, uh, I value out of it is the increased participation of, uh, people who are living with HIV who directly participate in research studies. It’s, uh, this study seems to have, uh, more, um, input than a lot of other studies that I’ve worked with, in terms of the research participation and the participants themselves, and I think that’s very rewarding to me.* | Rapid Autopsy Team |
|  | *There may be have some surreal moments and just thinking about what [the autopsy] means for this person and for the family and for the rest of us. Um, I think we do a really good job at taking care of each other after that act, which makes it nicer.* | Rapid Autopsy Team |
|  | *[Autopsy] is kind of hard and I think I can do it because of the great team and they know me…they know when sometimes I need to stop and take a break because it’s very hard for me to just put all my emotion on the side during the process. But I do it because of what [the participants] are doing for the study.* | Rapid Autopsy Team |
|  | *I really enjoy the preparation leading up to autopsy, the organized chaos that is the autopsy room, and then the, kind of, steady decline after when we just kind of relax in the lab with a little bit of pizza and probably wine [background laughter, chuckling]. But no, I think we really do work well because, I mean, we’re kind of like a family in the lab.* | Rapid Autopsy Team |
|  | *[I]t always makes me sort of proud and happy to see everybody working as a team [chuckling] in this completely chaotic, uh, mess that is completely organized to get everything done.* | Rapid Autopsy Team |
|  | *[T]he stress in the autopsy room is really minimized just because…I trust the people that I’m with. I know that if we don’t have enough tubes, we’ll figure it out, I know if something comes up that we’re not aware of, that we’ll figure it out, that we’ll work through it, and…and it’s going to be okay.* | Rapid Autopsy Team |
| **Considerations for Study Scale-Up at Other Clinical Research Sites** | | |
|  | *I think the research support is huge. I mean, to have the kind of team that we have on standby doing the autopsy is not easy to replicate, and so that will be very important.* | Clinical Team |
|  | *Our technicians are not normal set of technicians, like for some reason, I think it’s a mix of leadership and loyalty. They are just ready to go above and beyond what’s in their job description… They are proud and happy to be a part of it. I think that was really good, good leadership, good selling to them, making them feel a part of the bigger project, which I’m not sure everybody can replicate without that kind of relationship that [name] more than anybody else was able to carry it with the team, um…even in the lab.* | Clinical Team |
|  | *I was just in a meeting on age-related HIV neurodegeneration at the NIH last week, and people were talking about this study. Like, we [inaudible] but we need to be doing this across the board, and it’s like, ‘well, easier said than done but yes, you’re right.’* | Clinical Team |
|  | *I think it’s important for us to put together some kind of, um, “this is how you do it” guide for people, and this is how you engage the community and bring the ethicists in, and all the elements that we have, um, you know, managed to put together very successfully so they could do that as well. But I think getting buy-in from the local community is going to be really…that’s going to be the harder part, because that’s not the same everywhere.* | Clinical Team |
|  | *[T]he rapid autopsy part is…a very valuable part, but it’s also the hardest part of the entire study, because, of course, like being ready and making it work requires so much coordination from so many people. I don’t actually think we could do it with any other team, and this worries me a little bit that the Last Gift might not be so easily reproducible everywhere because I think the team is so unique. And the stress level during the autopsy is huge for everyone, and I recognize that. So, I’m extremely proud of what we put together.* | Rapid Autopsy Team |
